# The involvement of the low-oxygen-activated locus of *Burkholderia cenocepacia* in adaptation during cystic fibrosis infection

**DOI:** 10.1038/s41598-018-31556-6

**Published:** 2018-09-06

**Authors:** Louise Cullen, Andrew O’Connor, Sarah McCormack, Rebecca A. Owens, Giles S. Holt, Cassandra Collins, Máire Callaghan, Sean Doyle, Darren Smith, Kirsten Schaffer, David A. Fitzpatrick, Siobhán McClean

**Affiliations:** 1 Centre of Microbial Host Interactions, Institute of Technology Tallaght, Dublin, 24 Ireland; 2 School of Biomolecular and Biomedical Sciences, University College Dublin, Belfield, Dublin, 4 Ireland; 3 Department of Biology, Maynooth University, Co. Kildare, Ireland; 4 Faculty of Health and Life Sciences, Northumbria University, Newcastle upon Tyne, England; 5 Department of Microbiology, St. Vincent’s University Hospital, Elm Park, Dublin, Ireland

## Abstract

Chronic infection with opportunistic pathogens including *Burkholderia cepacia* complex (Bcc) is a hallmark of cystic fibrosis (CF). We investigated the adaptive mechanisms facilitating chronic lung infection in sequential Bcc isolates from two siblings with CF (P1 and P2), one of whom also experienced intermittent blood-stream infections (P2). We previously showed increased lung cell attachment with colonisation time in both P1 and P2. WGS analysis confirmed that the isolates are closely related. Twelve genes showed three or more mutations, suggesting these were genes under selection. Single nucleotide polymorphisms (SNVs) in 45 regulatory genes were also observed. Proteomic analysis showed that the abundance of 149 proteins increased over 61-months in sputum isolates, and both time- and source-related alterations in protein abundance between the second patient’s isolates. A consistent time-dependent increase in abundance of 19 proteins encoded by a low-oxygen-activated (lxa) locus was observed in both sets of isolates. Attachment was dramatically reduced in a *B*. *cenocepacia* K56-2Δlxa-locus deletion mutant, further indicating that it encodes protein(s) involved in host-cell attachment. Time-related changes in virulence in *Galleria mellonella* or motility were not observed. We conclude that the lxa-locus, associated with anoxic persistence *in vitro*, plays a role in host-cell attachment and adaptation to chronic colonization in the hypoxic niche of the CF lung.

## Introduction

Chronic infection is a hallmark of cystic fibrosis (CF) related lung disease. Several opportunistic pathogens, such as *Pseudomonas aeruginosa* and *Burkholderia cepacia* complex (Bcc) colonise the airways of CF patients impacting significantly on the quality of life and mortality of people with CF. Bcc is a group of 22 genetically distinct, highly antibiotic resistant bacterial species^1–4^ associated with a more dramatic decline than *P*. *aeruginosa*^5,6^. About 20% of Bcc colonised patients experience bacteraemia and “cepacia syndrome”, characterised by a sometimes fatal, necrotising pneumonia^7^. *B*. *cenocepacia* is the most virulent Bcc species and although most frequently associated with bacteraemia, this complication has also been linked to other Bcc species^5,8–10^.

Although the environmental reservoirs of Bcc infection are not fully elucidated, extensive isolation of CF patients has limited patient-to-patient transmission with the consequence that in recent years, many Bcc infections are acquired from the environment. Bcc has been isolated in a range of settings including the rhizosphere^11^ and disinfectants^12^ and has an impressive propensity to adapt to a range of environmental conditions. In response to the selection pressures of the host and antimicrobial therapies, bacterial pathogens must evolve to facilitate chronic colonisation^13,14^. Much of the research on bacterial adaptation in the CF context has focused on *P*. *aeruginosa*^13–16^, while the adaptive strategies of *B*. *cenocepacia* isolates have been examined to a lesser degree^17–20^. Reported consequences of *B*. *cenocepacia* adaptation include increased antimicrobial resistance, loss of motility, tolerance of iron limitation and increased virulence to host cells over time of chronic infection. In contrast, *P*. *aeruginosa* and *B*. *multivorans*, which is currently the most frequently isolated Bcc species in CF patients, showed reduced virulence over time of infection^21,22^. The mechanisms by which Bcc can adapt have not been elucidated to date.

We recently showed that two series of sequential isolates from two adult male siblings with CF (referred to as P1 and P2) increased their ability to attach to host epithelial cells over time of colonisation^23^. We conducted whole genome sequencing on six of these isolates (3 per patient) and an in-depth proteomic analysis. We were particularly interested in examining the mechanisms contributing to the increased host attachment over time. Previously, Sass *et al*.^24^, identified a 50-gene cluster in *B*. *cenocepacia* that was highly upregulated under low oxygen culture conditions (*lxa-*locus) and was required for persistence under anoxic conditions. This is significant as there are clear zones of hypoxia within the CF lung, predominantly caused by oxygen consumption by host immune cells, particularly polymorphonuclear leukocytes^25^. We now show that proteins encoded on the *lxa-*locus are consistently upregulated across both sets of patient isolates over time, highlighting that this gene cluster is relevant *in vivo* and likely to be important for niche adaptation to the hypoxic CF lung.

## Results

### Overview of whole genome sequencing of sequential *B*. *cenocepacia* isolates

Initial MLST of the sequential isolates used in this study (Supplementary information, Table S1) determined that these *B*. *cenocepacia* isolates all shared the same unique sequence type (ST867)^23^. Individual isolates were assembled *de novo*. Average coverage ranged from 23X(P2 late blood isolate, P2B3) to 171X(P2 sputum isolate, P2S) and the N50 scores ranged from 104238 (P2B3) to 246069 (P1 middle isolate, P1M). Total assembly lengths were approximately 7.7–7.8 million base pairs for individual species (Supplemental Table S1). We also mapped raw sequence reads against the *B*. *cenocepacia* J2315 reference genome, as these sequential ST867 isolates are in the same *recA* group as J2315 based on multi-locus sequence typing (MLST) sequence type. In addition, the J2315 genome is complete, well annotated^26^, actively curated (www.burkholderia.com)^27^ and a widely studied Bcc strain. A mean of 3.08 million reads were produced per isolate of which a mean of 88.31% (87.3 to 89.1%) were mapped to the J2315 reference with a mean coverage of 86.45% of the reference.

**Figure 1:**
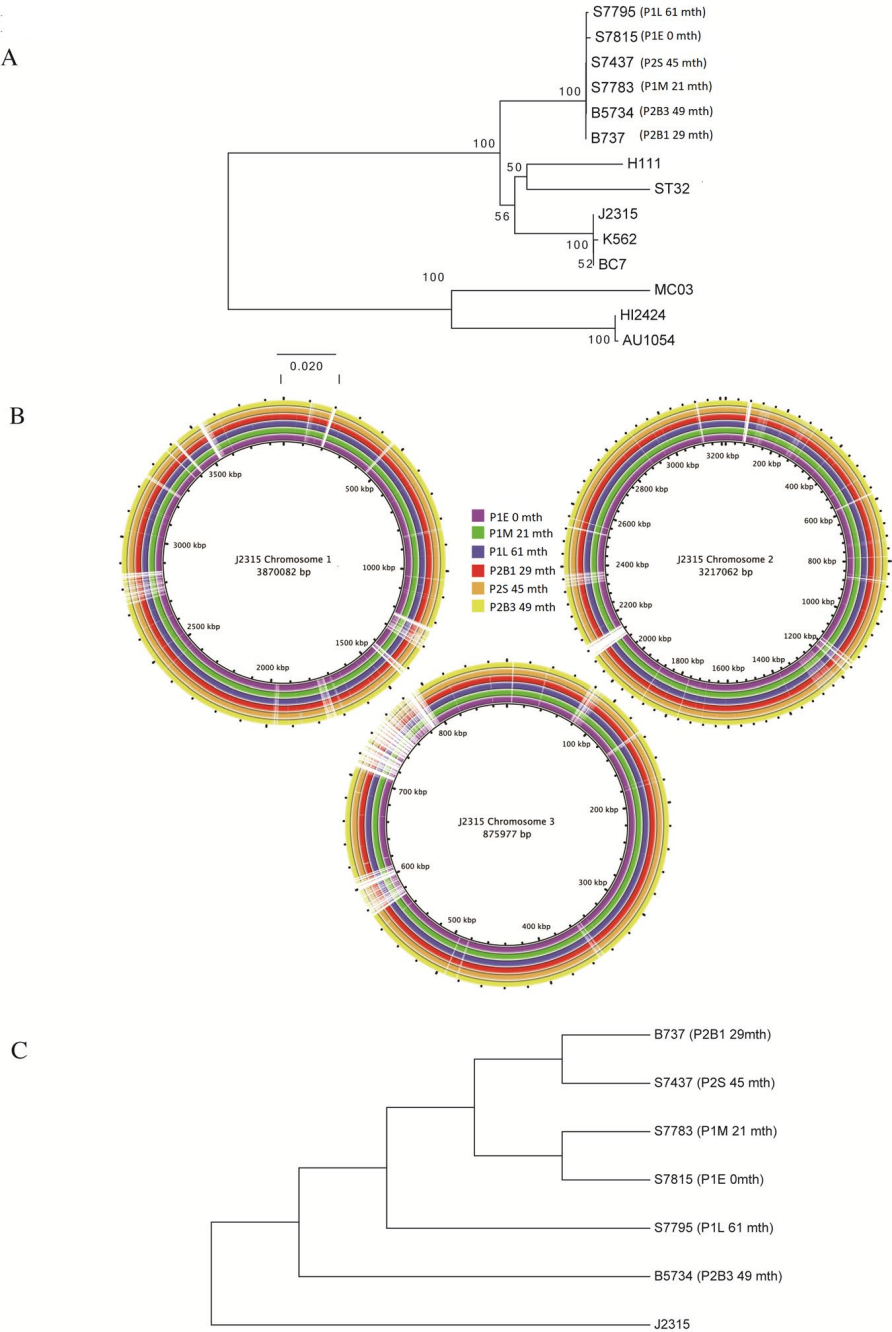
(**A**) Phylogenetic relationship amongst 14 *B*. *cenocepacia* isolates including our six of interest. The phylogeny was inferred using the Neighbor-Joining method as described in the methods. Bootstrap scores are shown at internal nodes. Branch lengths correspond to number of base substitutions per site. (**B**) Comparison of the genome sequences of the isolates with reference genome of *B*. *cenocepacia* strain J2315 (not drawn to scale) created using BRIG. (**C**) Phylogenetic relationship amongst our six *B*. *cenocepacia* isolates of interest. *B*. *cenocepacia* J2315 is included as an outgroup. The phylogeny was inferred using the Maximum Parsimony method as described in the methods. Bootstrap scores are shown at internal nodes. Input character data corresponded to all 1,132 SNVs called by both GATK and mpileup variant callers.

Comparing the concatenated MLST gene fragments from our isolates with other strains clustering under the same *B*. *cenocepacia recA* subgroup to construct a MLST based neighbour-joining phylogenetic tree, confirmed that the ST867 isolates are closely related to *recA* subgroup of strains (Fig. 1A). Comparative analysis of the assembled sequences using BLAST Ring Image Generator (BRIG) indicated that comparable to previously reported *B*. *cenocepacia* CF isolates, several genomic islands appeared to be absent among the ST867 isolates, namely BcenGI2, BcenGI3, BcenGI6, BcenGI9, BcenGI10, BcenGI13. Furthermore our sequences had poor coverage in other genomic islands, e.g. BcenG15, BcenGI7, BcenG18, BcenGI12 and BcenGI14 (Fig. 1B) as has been previously shown for other patient isolates^26,28^. Obvious differences, including insertions and deletions of significant portions of genomic material, were not apparent over time of chronic lung infection from patient 1 isolates or between the blood and sputum associated isolations from patient 2 based on the BRIG comparison to *B*. *cenocepacia* J2315 (Fig. 1B). With respect to the *B*. *cenocepacia* J2315 reference genome 57,050 and 62,532 single nucleotide polymorphisms (SNVs) across our six isolates were located using Genome analysis toolkit (GATK) and mpileup variant callers respectively. Of these 48,263 and 55,093 were found in all six isolates. From the remaining non-intersecting SNVs we identified 1,132 variants that were called by both GATK and mpileup. We took a conservative approach and only considered the SNVs located by both variant callers as *bona fide*.

Examining the 1132 variants across the six isolates, 976 (88%) were within genes. In total, of the 823 SNVs in coding regions, 528 (64%) were non-synonymous (NS) SNVs, while 36% were synonymous, the remaining variants were stop loss or stop gain mutations, indels and non-coding transcript variants. Based on the confident identification SNVs, we constructed a phylogenetic tree (Fig. 1C) to determine the relationships among the isolates. The early and middle isolates from P1 (P1E and P1M) are likely to share a common ancestor, as expected. In addition, the late isolate from P1 (P1L) and the third blood isolate from P2 (P2B3) have evolved into a separate clade, based on SNVs and putative internal node observed (Fig. 1C). From this tree it appears that P2B1 and P2S1 are more closely related to each other than each is to either P1E and P1M or P1L and P2B3. A full list of all SNVs and indels observed in these isolates relative to the *B*. *cenocepacia* J2315 is provided in the Supplementary Information Table S2. The average evolutionary rate was 3.50 × 10^−6^ SNVs/bp/year in the brothers isolates, which is higher than that observed in other studies, for example 5.3 × 10^−7^ SNV/bp/year in Canadian CF *B*. *cenocepacia* isolates^28^. The high evolutionary rate may be the result of a nonsynonymous SNV in the mismatch repair gene *mutS* shared by P1L and all P2 isolates which conferred a hypermutation phenotype. The mutation rate was 1.1 × 10^−6^ SNV/bp/year in the first patient prior to acquisition of the *mutS* mutation. The average mutation rate following the acquisition of the *mutS* mutation increased dramatically by three-fold in the later isolates, including all isolates from P2 which was first identified 8 months after the P1M isolate which lacked the *mutS* mutation.

Genes with multiple mutations or those which are fixed in the population are considered to be under selection^29^. There were 75 genes which had multiple mutations indicating that these genes were under strong selection pressure (Supplementary Information Table S2). Among these were 50 genes in which missense or frameshift mutations would result in an alteration of the gene product. There were 12 genes with three or more mutations (Table 1) which has a zero probability of occurring by chance^29^. In particular, the flagellar hook length control protein gene *fliK* showed 5 independent NS mutations, three in P2B3 and two in P2B1 which were unique to the individual isolates. There were 15 NS mutations in the BCAL1165 gene (which encodes Type VI secretion system base plate protein), identified in the P2S sample. This gene seemed highly mutable with 107 NS and S mutations in total across the six isolates. A total of eight independent mutations were observed in the proline transporter protein BCAL1252, seven of which were in P1L isolate. The ornibactin synthesis genes also found to have several mutations. The orbI gene showed 18 independent NS mutations all in the P2S isolate, while the orbJ gene showed 9 NS mutations, one of which was unique to P2S and the remainder shared by P2S and P2B3 and therefore fixed over time in this patient’s isolates. There were nine independent NS SNVs in the capsular polysaccharide transport gene (BCAM0209) in both late isolates, indicating selection pressure on this gene. Five of the mutations would result in substitutions of serines at three positions (Ser 52, Ser 47 and Ser 22). SNVs were also identified in 45 genes encoding for regulatory proteins, of which eight were fixed in later isolates, including lysR regulatory family protein genes (BCAL0707; BCAM0056; BCAM0404), two-component regulatory systems genes (BCAM0714) and TetR family regulatory protein gene (BCAS0083) (Table S2). The Fis family transcriptional regulator BCAM0871 showed two mutations, a NS SNV (Arg to Cys) and a frameshift at Gly 321, suggesting it is also a gene under selection. Interestingly, all regulatory gene SNVs were absent from P1M (using P1E as the reference), consistent with the appearance of the mutS mutation (Table S2). In order to validate the WGS sequencing, two genes were selected, amplified and the SNPs confirmed by Sanger sequencing. The missense variant at position 205 (G > A) in P2B1, was confirmed in BCAM0292 (Supplementary Fig. S1A); the synonymous variant at position 222 (G > A) was confirmed in P2B1 and P2S and the missense variant at position 789 (C > T) in P2S (Supplementary Fig. 2B) was confirmed in BCAL1700 (Supplementary Fig. S1B).

**Table 1:** Genes with three or more SNVs in the *B*. *cenocepacia* sequential isolates.

Location	Gene product	Mutation Category	Type of mutation	Presence	Effect in protein
AM747720_581699 AM747720_ 581800 AM747720_ 581817 AM747720_ 582072 AM747720_ 582104	FliK, flagellar hook length control protein BCAL0529	CDS	5 nonsyn SNP	P2 blood isolates only	p.Pro200Thr p.Glu233Asp p.Leu239Pro p.Gly324Asp p.Gly335Arg
AM747720_1270941 AM747720_1270992 AM747720_1271269 AM747720_1271301 AM747720_1271327 AM747720_1271523 AM747720_1271597 AM747720_1271649 AM747720_1271651 AM747720_1271655 AM747720_1271661 AM747720_1271662 AM747720_1271883 AM747720_1272037 AM747720_1272747	BCAL1165, Type VI secretion base plate	CDS	15 nonsyn SNP	P2 Sputum isolate only	p.Val136Ile p.Thr153Ala p.Val245Ala p.Thr256Ala p.Asp264Glu p.Ala330Thr p.Met354Ile p.His372Asn p.His372Gln p.Thr374Ala p.Val376Ile p.Val376Ala p.Thr450Ala p.Val501Ala p.Ser738Pro
AM747720_1361546 AM747720_1361548 AM747720_1361552 AM747720_1361554 AM747720_1361566	Putative proline betaine transporter, BCAL1252	CDS	5 nonsyn SNP		p.Met234Ile p.Met234Leu p.Ile232Met p.Ile232Val p.Ile228Val
AM747720_1858993 AM747720_1859407 AM747720_1859523 AM747720_1860079 AM747720_1860163 AM747720_1860568 AM747720_1860791 AM747720_1861173 AM747720_1861423 AM747720_1861426 AM747720_1861604 AM747720_1862398 AM747720_1862506 AM747720_1862562 AM747720_1862590 AM747720_1863283 AM747720_1863729 AM747720_1863879	Ornibactin synthesis, orbI	CDS	18 nonsyn,	P2 sputum isolate	p.Gly1174Asp p.Gly1312Asp p.Val1351Phe p.Ala1536Glu p.Ala1564Gly p.Ala1699Val p.Asp1773Glu p.Ser1901Ala p.Ala1984Glu p.Ala1985Glu p.Asp2044Glu p.Arg2309His p.His2345Arg p.Ala2364Thr p.Ala2373Val p.Ile2604Thr p.Val2753Ile p.Arg2803Gly
AM747720_ 1867064 AM747720_ 1866582 AM747720_1866589 AM747720_1868223 AM747720_1868665 AM747720_1869043 AM747720_1869058 AM747720_1869639 AM747720_1869880	orbJ	CDS	2 FS, 7 nonsyn	P2S only All in P2 sputum and late blood	p.Leu639fs p.Ser478fs p.Pro480Leu p.Thr1025Ala p.Ala1172Val p.Gln1298Arg p.Leu1303Pro p.Ala1497Thr p.Ala1577Val
AM747720_1917231 AM747720_1917465 AM747720_1917861 AM747720_1917341	Putative universal stress protein BCAL1736		Insertion 2FS 1 nonsyn	P2B1 and P2B3 P1B2 only P1L P2B3	p.Gly80_His81insGlnProGlyAlaGly p.Gly156fs p.Ala289fs p.Val113Ala
AM747720_2835980 AM747720_2835981 AM747720_2836103	Putative transmembrane component of ABC transporter BCAL2576	CDS	3 nonsyn	all P2B3	p.His26Tyr p.His26Pro p.Leu67Phe
AM747721_246833 AM747721_246838 AM747721_246845 AM747721_246847 AM747721_246848 AM747721_246850 AM747721_246874 AM747721_246923 AM747721_246953	BCAM0209 capsular polysaccharide transport	CDS	9 nonsyn		p.Ser52Ala p.Val50Gly p.Thr48Ala p.Ser47Phe p.Ser47Pro p.Gln46Pro p.Met38Thr p.Ser22Ala p.Thr12Ala
AM747721_391712 AM747721_392768 AM747721_392870	Hypothetical conserved protein BCAM0337	CDS	3 nonsyn	P2B3 P2B3 P2S	p.Arg66His p.Gln418Arg p.Tyr452Cys
AM747721_1514436 AM747721_1514440 AM747721_514440	Putative penicillin binding protein, BCAM1362		2FS, 1 nonsyn	P1L P2 all three isolates P1L	p.Pro624fs p.Ala623fs p.Ala623Pro
AM747722_560968 AM747722_560969 AM747722_560969	Cytochrome C family protein, BCAS0491		2FS, 1 nonsyn	P1L P2B1, P2S P1M	p.Ser96fs p.Ser98fs p.Ser96Pro
AM747720_2426852 AM747720_2427052 AM747720_2427212	BCAL2199, transcriptional regulator protein	CDS	1FS, 2 nonsyn	P1L, P2B3 P2S P2B1	p.Ala145fs p.Asp78Gly p.Pro25Ser

#### B. cenocepacia ST867 proteome alterations over time of colonization

We previously demonstrated an increase in host cell attachment in both series of isolates from the two patients, which was independent of the location of infection^23^. Our primary aim was to identify global changes in bacterial protein abundance over time of colonization that may contribute to this increased host cell attachment. A comprehensive proteomic study was performed on the six isolates. Two types of data were obtained: (A) differentially abundant proteins with >1.5-fold alteration between isolates and (B) uniquely detected proteins, i.e. proteins whose presence was significantly increased from, or decreased to, non-detectable levels. The total numbers of altered proteins in each comparison are listed in Supplementary Information Table S3.

#### Proteins that altered over time of colonization in P1

Given that proteins are the functional molecules in cells, investigating alterations in protein abundance is likely to be a more informative indicator of phenotype alterations. Despite the high level of SNVs observed, there were only 149 proteins that showed a statistically significant (P < 0.05) increased abundance by >1.5-fold over the 61-month period of chronic lung infection examined for patient 1 (Supplementary Information Table S4). Differentially abundant proteins of particular interest are highlighted in Table 2. Consistent with the previously observed increase in attachment over time of colonization, a substantial number of cell surface-associated proteins were significantly increased from P1E to P1L including: trimeric autotransporter adhesin (TAA) (BCAM0219); fimbrial usher pilus protein (BCAL1828); WbxY, involved in O-antigen biosynthesis, and BCAL3149, an outer membrane lipoprotein-sorting protein. Two alkylhydroperoxide reductases, (AhpC and AhpD), involved in responses to oxidative stress, showed increased abundance in P1L relative to P1E. We have previously identified AhpC as being involved in host cell attachment^30^.

**Table 2:** Examples of differentially abundant proteins detected in the sequential sputum isolates over time of chronic infection.

Protein	Gene ID J2315^a^	Comparison	Up/Down	Fold change	t-test P value	Sequence coverage [%]^b^	PI^c^	Mol. weight [kDa]^d^	Function^e^
Putative type VI secretion system protein TssF	L0345	E → L	Down	1.54	0.024	11.45	6.51	68.84	Pathogenesis/Intracellular trafficking, secretion, Type VI protein secretion system component VasA
Putative type VI secretion system protein TssG	L0346	E → L	Down	1.75	0.049	21.74	5.70	40.29	Pathogenesis/Intracellular trafficking, secretion, Type VI protein secretion system component
Putative Fur family transcriptional regulator	L2812	E → L	Down	5.92	0.000	32.70	6.22	16.83	Iron regulation
		M → L	Down	4.18	0.000	35.13			
Putative periplasmic solute-binding protein	L2813	E → L	Down	3.17	0.004	60.18	7.11	32.45	ABC metal ion transport system, periplasmic component/surface adhesin
Putative cytidylyltransferase	L0691	E → L	Up	1.66	0.003	39.68	6.29	17.46	Biosynthesis and degradation of surface polysaccharides and lipopolysaccharides
Ornibactin biosynthesis ABC transport protein	orbE L1695	E → M	Up	8.12	0.000	5.40	7.76	62.70	Iron assimilation/ornibactin biosynthesis
Putative fimbrial usher protein	L1828	E → L	Up	2.99	0.001	3.25	10.07	85.88	Cell motility and secretion/Intracellular trafficking and secretion/P pilus assembly protein, porin PapC
Alkyl hydroperoxide reductase AhpD	ahpD L2014	E → L	Up	1.59	0.003	48.39	5.93	18.77	Detoxification
Uncharacterized protein	wbxY L3111	E → L	Up	2.32	0.038	34.70	8.44	32.74	LPS biosynthesis/O-antigen biosynthetic process
Putative exported protein	L3149	E → L	Up	14.66	0.000	25.83	5.97	22.72	Cell envelope biogenesis, outer membrane/Outer membrane lipoprotein-sorting protein
		M → L	Up	1.99	0.004	39.53			
CRP family regulatory protein	M0049	E → L	Up	3.98	0.000	61.50	7.67	27.20	Signal transduction/cAMP binding protein, activator and regulator of cAMP dependent proteases
		E → M	Up	1.92	0.002	51.30			
		M → L	Up	1.87	0.000	60.00			
Putative haemagluttinin-related autotransporter protein	M0219	E → L	Up	2.01	0.028	12.61	4.57	288.73	Trimeric autotransporter/YadA-like C terminal
Universal stress protein	M0276	E → L	Up	6.01	0.001	26.99	7.82	17.02	UspA family stress protein
		E → M	Up	3.67	0.007	24.09			
		M → L	Up	1.76	0.016	30.98			
Uncharacterized protein	M0277	E → L	Up	6.32	0.001	60.30	6.89	10.26	unknown function DUF1488
		E → M	Up	2.85	0.014	40.15			
		M → L	Up	2.22	0.013	62.90			
Putative heat shock protein	M0278	E → L	Up	40.47	0.000	35.69	4.78	15.81	chaperone/post translational modification
		E → M	Up	4.35	0.001	20.65			
		M → L	Up	3.03	0.002	36.65			
Putative phospholipid-binding protein	M0280	E → L	Up	30.48	0.000	31.94	5.98	23.52	Predicted periplasmic or secreted lipoprotein/Bon domain
		E → M	Up	4.94	0.010	17.83			
		M → L	Up	4.73	0.000	41.44			
Putative cytochrome c	M0284	M → L	Up	3.60	0.003	23.40	5.63	12.04	Energy production and conversion
Uncharacterized protein	M0285	E → L	Up	5.97	0.000	53.71	5.93	36.09	flavin or nitro compound reduction
		E → M	Up	3.29	0.000	56.06			
		M → L	Up	1.85	0.014	54.43			
Putative universal stress protein	M0290	E → L	Up	8.09	0.000	56.90	4.81	16.14	UspA family Stress protein
		E → M	Up	4.25	0.000	71.38			
		M → L	Up	2.00	0.001	74.43			
Putative universal stress protein	M0291	E → L	Up	13.43	0.000	68.93	6.26	30.52	USP stress family protein
		E → M	Up	5.52	0.000	54.83			
		M → L	Up	2.42	0.000	74.15			
Putative universal stress protein	M0292	E → L	Up	16.74	0.000	37.71	5.05	17.74	UspA family stress protein
		E → M	Up	6.75	0.000	22.80			
		M → L	Up	2.49	0.002	39.20			
Putative universal stress protein	M0294	E → L	Up	10.91	0.000	60.79	6.26	30.59	UspA family stress protein
		E → M	Up	4.94	0.000	57.51			
		M → L	Up	2.49	0.000	62.39			
Uncharacterized protein	M0295	E → L	Up	2.90	0.006	37.43	5.65	19.82	hypothetical protein
		M → L	Up	2.10	0.011	44.00			
Acetoacetyl-CoA reductase	phbB M0296	E → L	Up	23.17	0.000	61.90	6.50	26.32	Secondary metabolite biosynthesis
		E → M	Up	6.46	0.000	56.36			
		M → L	Up	2.85	0.000	69.89			
Putative phosphate acetyl/butyryl transferase	M0298	E → L	Up	17.20	0.000	26.79	5.79	35.26	Energy production and conversion
		E → M	Up	6.02	0.000	29.25			
		M → L	Up	1.97	0.001	35.65			
Putative zinc-binding alcoholdehydrogenase	M0299	E → L	Up	8.64	0.000	40.98	6.13	36.93	Amino acid transport and metabolism/threonine 3- dehydrogenase activity
		E → M	Up	3.99	0.005	40.23			
		M → L	Up	2.47	0.001	45.41			
Metallo-beta-lactamase superfamily protein	M0300	E → L	Up	23.94	0.000	64.09	6.30	50.96	Translation
		E → M	Up	5.85	0.000	57.24			
		M → L	Up	3.20	0.000	74.30			
Uncharacterized protein	M0308	E → L	Up	8.02	0.000	43.36	8.93	19.46	hypothetical protein
		E → M	Up	3.02	0.001	33.04			
		M → L	Up	2.55	0.000	47.29			
Phosphofructokinase	M0311	E → L	Up	6.55	0.000	26.03	7.58	32.33	Carbohydrate transport and metabolism
		E → M	Up	3.26	0.002	21.39			
		M → L	Up	2.91	0.000	41.13			
Uncharacterized protein	M0316	E → L	Up	14.10	0.007	37.98	7.06	16.85	Unknown
Putative universal stress protein	M0319	E → L	Up	7.56	0.000	39.54	6.36	33.62	UspA family stress protein
		E → M	Up	2.96	0.002	30.50			
		M → L	Up	1.77	0.015	50.99			
Putative aldobloodstream/keto reductase	M0356	E → L	Up	7.09	0.007	21.25	5.55	37.44	oxidoreductase/aldoketo reductase
Alkyl hydroperoxide reductase subunit C	ahpC M1217	E → L	Up	1.81	0.002	45.38	5.08	20.73	Adaptations to atypical conditions/peroxiredoxin activity
Uncharacterized protein	S0292	E → L	Up	2.36	0.001	87.98	4.81	19.21	Inclusion body protein PixA
		E → M	Up	1.74	0.011	85.88			
Nematocidal protein AidA	aidA S0293	E → L	Up	2.00	0.031	92.50	6.09	18.87	Nematocidal Virulence

E = early sputum isolate (P1E), M = middle sputum isolate (P1M) and L = late sputum isolate (P1L).

^a^Gene ID denoted by chromosome code, each letter L, M, S is preceded by BCA, i.e. L = BCAL; M = BCAM; S = BCAS.

^b^% of sequence covered by matching peptides for the identified protein in the database.

^c^pI isoelectric point, the pH at which the identified protein has no net charge, as determined by expasy.org (http://web.expasy.org/compute_pi/).

^d^Molecular weight as determined by Q-Exactive LC-MS and max quant relative quantitation using *B*. *cenocepacia* J2315 database.

^e^protein function as determined by searching the burkholderia.com database^27^.

For full list of differentially expressed proteins see Supplementary Information Table S5.

Of particular interest were a group of 19 proteins encoded within 50-gene cluster with significantly increased abundance over time of chronic infection. This cluster is designated as a low-oxygen-activated (*lxa*) locus (BCAM0275-BCAM0323) which was associated with persistence in *B*. *cenocepacia* J2315 when cultured in a limited oxygen environment^24^. These 19 proteins were substantially increased (up to 40-fold) over time of chronic lung infection in all comparisons in P1 (Table 2). Six universal stress proteins (USP) are encoded on this locus and all six USPs, together with a heat shock protein, showed increased abundance in the later isolates. A BON (bacterial OsmY and nodulation)-domain phospholipid-binding protein (BCAM0280) showed a 30-fold abundance increase over the 61-month period examined. Reduced abundance of 151 proteins over time of infection from the early to late sputum isolate from P1 was observed (Supplementary Information Table 4), including type VI secretion system (T6SS) proteins, TssF and TssG (Table 2), associated with virulence. Seventy-nine proteins were detected in one P1 isolate which were absent or undetectable in the other comparators (Supplementary Information Table 5), including penicillin binding protein, DacB and virulence factor, ZmpA^31,32^ which were both absent in the first two isolates. In contrast, another T6SS protein was only detected in P1E.

#### Proteins that altered over time of colonization in P2

Consistent with P1 isolates, there was also substantial time-associated increased abundance of 20 *lxa*-encoded proteins (Table 3) in the sequential P2 isolates, including the same 19 identified in P1 isolates with an additional uncharacterised protein (BCAM0307). Significantly the BON-domain phospholipid binding protein (BCAM0280) showed dramatically increased abundance (122-fold) in P2B3 relative to P2B1.

**Table 3:** Examples of differentially abundant proteins detected in P2 blood and sputum isolates over time of chronic infection.

Protein	Gene ID J2315^a^	Comparison	Up/Down	Fold change	t-test P value	Sequence coverage [%]^b^	PI^c^	Mol. Weight [kDa]^d^	Function^e^
Chemotaxis response regulator protein-glutamate methylesterase	cheB1 L0134	B1 → B3	Up	2.65	0.0016	26.20	8.44	38.92	chemotaxis/Cell Motility
		B1 → S	Up	2.70	0.0001	26.40			
Chemotaxis protein CheY	cheY L0135	B1 → B3	Up	3.30	0.0035	59.80	5.82	14.24	Signal transduction/chemotaxis
Putative type VI secretion system protein TssD	bcsL L0343	S → B3	Down	1.97	0.0336	91.00	6.70	18.42	type VI secretion system effector, Hcp1 family/intracellular trafficking, secretion, and vesicular transport/pathogenesis
		B1 → S	Up	1.71	0.0139	91.00			
Putative dioxygenase	orbG L1690	S → B3	Down	3.33	0.0090	30.10	4.83	37.39	Iron assimilation/ornibactin biosynthesis
Ornibactin synthetase F	orbF L1693	S → B3	Down	7.94	0.0001	38.70	5.74	30.81	Iron assimilation/siderophore-iron reductase
		B1 → S	Up	113.40	0.0000				
Ornibactin biosynthesis ABC transport protein	orbE L1695	S → B3	Down	4.54	0.0057	17.00	7.76	62.70	Iron assimilation/ornibactin biosynthesis
		B1 → S	Up	4.91	0.0006				
Putative fimbrial usher protein	L1828	B1 → B3	Up	1.69	0.0130	70.60	10.07	85.88	Cell motility and secretion/Intracellular trafficking and secretion/P pilus assembly protein, porin PapC
L-arabinose formyltransferase	L1933	S → B3	Down	1.53	0.0176	33.00	6.11	33.54	lipid A Biosynthesis and degradation of surface polysaccharides and lipopolysaccharides Protein synthesis/tRNA aminoacylation
		B1 → B3	Down	2.02	0.0010	23.00			
UDP-glucuronic acid decarboxylase	L1934	B1 → B3	Down	1.56	0.0012	47.80	5.76	39.45	Biosynthesis and degradation of surface polysaccharides and lipopolysaccharides
Outer membrane protein assembly factor BamA	bamA L2083	B1 → B3	Up	1.53	0.0294	58.20	8.42	84.99	Outer membrane protein
		B1 → S	Up	1.82	0.0151	11.70			
Putative glycosyltransferase	L2404	B1 → B3	Down	1.89	0.0006	68.10	9.47	45.10	Lipopolysaccharide biosynthesis
		B1 → S	Down	1.66	0.0037	26.20			
Putative OmpA family membrane protein	L2645	B1 → S	Up	1.61	0.0119	35.80	9.77	21.57	OmpA/MotB family protein
UDP-N-acetylenolpyruvoylglucosamine reductase	murB L2768	S → B3	Down	1.61	0.0001	27.10	6.11	37.56	Peptidoglycan biosynthesis
Putative periplasmic solute-binding protein	L2813	S → B3	Down	4.82	0.0006	63.70	7.11	32.45	ABC metal ion transport sytem, perilasmic component/surface adhesin
		B1 → B3	Down	27.68	0.0000	51.60			
		B1 → S	Down	5.25	0.0001	64.00			
Putative membrane protein	L2947	B1 → S	Up	2.26	0.0068	11.50	5.86	43.82	Lipopolysaccharide biosynthesis
Putative ompA family protein	L2958	S → B3	Up	1.54	0.0403	40.10	9.28	23.97	Cell envelope biogenesis, Outer membrane protein and related peptidoglycan-associated (lipo)proteins
Uncharacterized protein	wbxY L3111	B1 → S	Up	3.06	0.0054	44.20	8.44	32.74	LPS biosynthesis/O-antigen biosynthetic process
Putative TolQ transport transmembrane protein	tolQ L3200	B1 → S	Up	2.55	0.0051	20.40	9.68	24.99	Pathogenesis/Intracellular trafficking and secretion/biopolymer transport protein
Putative TolR-related protein	tolR L3201	B1 → S	Up	1.53	0.0392	26.80	9.34	16.01	Pathogenesis/Intracellular trafficking and secretion/biopolymer transport protein
Ferric uptake regulator	fur L3378	S → B3	Down	1.58	0.0000	71.80	5.96	16.23	Ferric uptake regulator
		B1 → S	Up	1.67	0.0000	71.80			
UDP-N-acetylmuramate:L-alanyl-gamma-D-glutamyl-meso-diaminopimelate ligase	mpl L3416	B1 → S	Down	38.26	0.0000	52.70	5.84	49.48	Cell envelope biogenesis, outer membrane/biosynthesis & degradation of murein and peptidoglycan
		S → B3	Up	34.48	0.0000	50.50			
Type II secretion system protein G	gspG L3523	B1 → B3	Up	1.75	0.0155	49.50	7.95	16.78	Cell motility and secretion/Intracellular trafficking and secretion/pathogenesis
		B1 → S	Up	2.33	0.0040	44.70			
CRP regulatory protein	M0049	S → B3	Up	1.54	0.001	67.70	7.67	27.20	Signal transduction/cAMP binding protein, activator and regulator of cAMP dependent proteases
		B1 → B3	Up	5.54	0.000	58.80			
		B1 → S	Up	3.33	0.000	69.00			
Putative haemagglutinin-related autotransporter protein	M0219	S → B3	Up	2.97	0.0005	6.70	4.57	288.73	Trimeric autotransporter/YadA-like C terminal
		B1 → S	Down	3.04	0.0115	7.20			
Universal stress protein	M0276	S → B3	Up	1.65	0.0010	58.30	7.82	17.02	UspA family stress protein
		B1 → B3	Up	6.74	0.0001	41.10			
		B1 → S	Up	3.89	0.0000	34.60			
Uncharacterized protein	M0277	S → B3	Up	1.61	0.0263	68.10	6.89	10.26	unknown function DUF1488
		B1 → B3	Up	3.46	0.0001	24.30			
		B1 → S	Up	1.92	0.0046	68.10			
Putative heat shock protein	M0278	S → B3	Up	6.67	0.0000	55.60	4.78	15.81	chaperone/post translational modification
		B1 → B3	Up	32.93	0.0000	31.60			
		B1 → S	Up	4.39	0.0002	24.30			
Putative phospholipid-binding protein	M0280	S → B3	Up	19.89	0.0000	53.20	5.98	23.52	Predicted periplasmic or secreted lipoprotein/Bon-domain
		B1 → B3	Up	122.12	0.0000	76.10			
		B1 → S	Up	3.70	0.0355	38.90			
Putative cytochrome c	M0284	S → B3	Up	2.54	0.0370	23.40	5.63	12.04	Energy production and conversion
		B1 → B3	Up	4.13	0.0071	19.60			
Uncharacterized protein	M0285	S → B3	Up	1.57	0.0052	65.40	5.93	36.09	flavin or nitro compound reduction
		B1 → B3	Up	3.03	0.0001	61.60			
		B1 → S	Up	1.93	0.0003	65.70			
Putative universal stress protein	M0290	S → B3	Up	1.64	0.0127	62.80	4.81	16.14	UspA family Stress protein
		B1 → B3	Up	4.34	0.0001	56.40			
		B1 → S	Up	2.79	0.0005	62.80			
Putative universal stress protein	M0291	S → B3	Up	2.15	0.0000	76.50	6.26	30.52	USP stress family protein
		B1 → B3	Up	3.87	0.0000	72.20			
		B1 → S	Up	1.65	0.0060	66.40			
Putative universal stress protein	M0292	S → B3	Up	2.10	0.0009	51.50	5.05	17.74	UspA family stress protein
		B1 → B3	Up	5.82	0.0000	69.50			
		B1 → S	Up	3.56	0.0000	22.80			
Putative universal stress protein	M0294	S → B3	Up	1.98	0.0014	64.90	6.26	30.59	UspA family stress protein
		B1 → B3	Up	3.30	0.0000	25.00			
		B1 → S	Up	1.68	0.0003	61.60			
Uncharacterized protein	M0295	S → B3	Down	1.84	0.0028	52.50	5.65	19.82	hypothetical protein
		B1 → B3	Up	3.93	0.0033	48.70			
		B1 → S	Up	10.07	0.0000	52.50			
Acetoacetyl-CoA reductase	M0296	B1 → B3	Up	4.37	0.0000	22.80	6.50	26.32	Secondary metabolite biosynthesis
		B1 → S	Up	1.54	0.0086	62.90			
		S → B3	Up	2.80	0.0003	72.60			
Putative phosphate acetyl/butyryl transferase	M0298	S → B3	Up	1.67	0.0105	42.00	5.79	35.26	Energy production and conversion
		B1 → B3	Up	4.62	0.0000	60.00			
		B1 → S	Up	2.18	0.0001	42.00			
Putative zinc-binding alcoholdehydrogenase	M0299	S → B3	Up	2.29	0.0027	48.30	6.13	36.93	Aminoacid transport and metabolism/threonine 3- dehydrogenase activity
		B1 → B3	Up	4.24	0.0000	37.80			
		B1 → S	Up	1.82	0.0188	43.40			
Metallo-beta-lactamase superfamily protein	M0300	S → B3	Up	1.54	0.0053	81.40	6.30	50.96	Translation
		B1 → B3	Up	8.31	0.0000	55.80			
		B1 → S	Up	4.76	0.0000	70.40			
Uncharacterized protein	M0307	S → B3	Up	4.67	0.0046	20.50	5.71	23.33	Unknown/Predicted periplasmic or secreted lipoprotein
Uncharacterized protein	M0308	S → B3	Up	1.76	0.0002	56.80	8.93	19.46	hypothetical protein
		B1 → B3	Up	2.87	0.0000	74.60			
		B1 → S	Up	1.53	0.0021	44.40			
Phosphofructokinase	M0311	S → B3	Up	1.82	0.0068	55.30	7.58	32.33	Carbohydrate transport and metabolism
		B1 → B3	Up	5.52	0.0000	37.70			
		B1 → S	Up	2.66	0.0003	53.10			
Uncharacterized protein	M0316	B1 → B3	Up	2.26	0.0380	24.90	7.06	16.85	Unknown
Putative universal stress protein	M0319	B1 → B3	Up	7.98	0.0001	23.40	6.36	33.62	UspA family stress protein
		B1 → S	Up	5.42	0.0001	58.20			
N-acylhomoserine lactone dependent regulatory protein CepR	cepR M1868	B1 → B3	Up	1.66	0.0404	70.80	5.60	26.61	Signal transduction mechanisms/Transcription/positive regulation of single-species biofilm formation/intraspecies QS/bacterial-type flagellar swarming motility
		B1 → S	Up	1.62	0.0058	19.70			
Nematocidal protein AidA	aidA S0293	B1 → S	Down	1.87	0.0151	91.00	6.09	18.87	Virulence
		S → B3	Up	1.64	0.0108	91.00			

B1 = P2 first blood isolate (P2B1), S = P2 sputum isolate (P2S) and B3 = P2 third blood isolate (P2B3).

^a^Gene ID denoted by chromosome code, each letter L, M, S is preceded by BCA i.e. L = BCAL; M = BCAM; S = BCAS.

^b^% of sequence covered by matching peptides for the identified protein in the database.

^c^pI isoelectric point, the pH at which the identified protein has no net charge, as determined by expasy.org (http://web.expasy.org/compute_pi/).

^d^Molecular weight as determined by Q-Exactive LC-MS and max quant relative quantitation using *B*. *cenocepacia* J2315 database.

^e^protein function as determined by searching the burkholderia.com database^27^.

For full list of differentially expressed proteins see Supplementary information Table S5.

These isolates had also previously shown increased attachment to lung epithelial cells over time of colonisation, despite the later isolate being a blood isolate^23^. Consistent with this, many time-dependent alterations in cell surface-related proteins were observed, including proteins involved in LPS synthesis: L-arabinose formyltransferase, UDP-glucuronic acid decarboxylase and a glycosyltransferase which showed reduced abundance over time. In contrast, WbxY, involved in O-antigen synthesis was increased 3-fold from P2B1 to P2S. Fimbrial usher protein and BamA were also elevated in the P2B3 blood isolate relative to P2B1. The quorum sensing (QS) regulatory protein CepR, was elevated in abundance in both P2S and P2B3 relative to P2B1, while the abundance of three proteins involved in chemotaxis and motility (CheB1, CheY and GspG) were also increased over time from P2B1 to P2B3.

Although only one sputum isolate was available for comparison, when P2 blood and sputum isolates were compared for source-dependent changes, obvious differences between cell-surface associated protein abundance were also observed. Mpl, involved in peptidoglycan synthesis and degradation, was strongly increased (34- to 38-fold) in P2S relative to both blood isolates (Table 3). TAA (BCAM0219) was elevated in both blood isolates relative to P2S. Other cell surface-related alterations include two putative OmpA family proteins (BCAL2645 and BCAL2958), which were higher in P2S relative to both blood isolates. Other proteins that were differentially abundant between blood and sputum isolates include a T6SS protein, BcsL, elevated in P2S relative to both of the blood isolates. Increased abundance of iron acquisition-associated proteins was also seen in P2S relative to blood isolates, including FUR, OrbF, OrbE and OrbG. Conversely, nematocidal protein, AidA, showed increased abundance in both blood isolates relative to P2S.

In total there were 125 proteins that changed from being undetectable to detectable among the three P2 isolates (Supplementary Information Table S5). Both time-dependent and site of isolation-related alterations were apparent. QS-associated protein, BCAM0188, involved in virulence factor regulation, was only detected in the P2B3 isolate while the virulence factor, ZmpB^33^, was only detected in the P2B1 and P2S isolates (Supplementary Information Table S5). Transmembrane protein, TolA and T6SS protein (BCAM0043) were only detected in the P2S and P2B3 isolates relative to P2B1 and consequently were time-dependent alterations. In contrast, iron acquisition proteins (OrbA and OrbB), OmpW and penicillin-binding protein, DacB, were not detected in either blood isolate but were present in P2S, indicating adaptations to blood.

#### Confirmation of up-regulation of selected genes

In order to substantiate the alterations in protein abundance, we examined the expression of genes encoding two consistently elevated *lxa*-encoded proteins, BCAM0280 and BCAM0276 by qPCR. Consistent with the proteomic analysis, a time-dependent increase in gene expression was observed for BCAM0280 and BCAM0276 genes from all six P1 and P2 isolates in all comparisons (Supplementary Information Table S6). This suggests that the observed protein adaptations were the result of altered gene expression over time of colonization, rather than due to altered protein processing. In addition, late in our study we obtained an additional isolate from P1 which was isolated 90 months after P1E (and which we designated P190) and a second sputum isolate from P2 isolated 64 months after P1E. qPCR analysis of these additional late isolate also confirmed the two genes BCAM0280 and BCAM0276 were up-regulated over time in these two late additional isolates (Supplementary Table S6).

#### *Virulence of* B. cenocepacia *isolates in* Galleria mellonella

**Figure 2:**
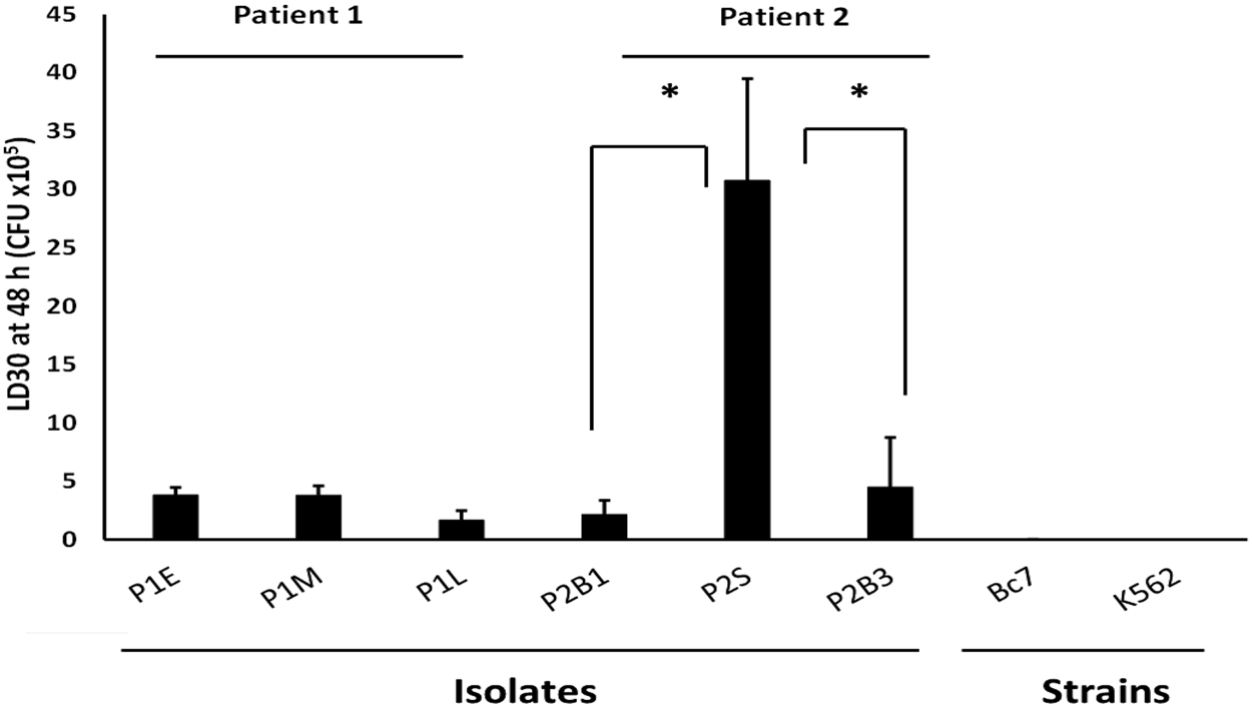
Virulence of the sequential sputum isolates from P1 and blood and sputum isolates from P2 in the *G*. *mellonella* infection model. Bars represent the mean LD_30_ values at 48 hours, determined on three independent occasions. Error bars represent standard error of the mean; *P < 0.05 as determined by ANOVA.

In order to examine whether the alterations in proteomic profile resulted in other consistent phenotypic changes across the two series of isolates, virulence was examined. All six ST867 isolates examined were considerably less virulent than both positive control strains in the *G*. *mellonella* infection model and LD_50_ values (CFU that resulted in 50% death of the larvae) could not be determined for every isolate at 48 h or 72 h. Consequently LD_30_ values (CFU that resulted in 30% death of the larvae) at 48 h were used to enable comparisons of all isolates. The LD_30_ values were comparable across P1 isolates (P = 0.084). Both blood isolates (P2B1 and P2B3) were 7- to 14-fold more virulent than P2S (Fig. 2) (P < 0.05), consistent with an adaptation between blood and lung in this patient.

#### Cytokine responses of CF epithelial cells to sequential B. cenocepacia isolates

**Figure 3:**
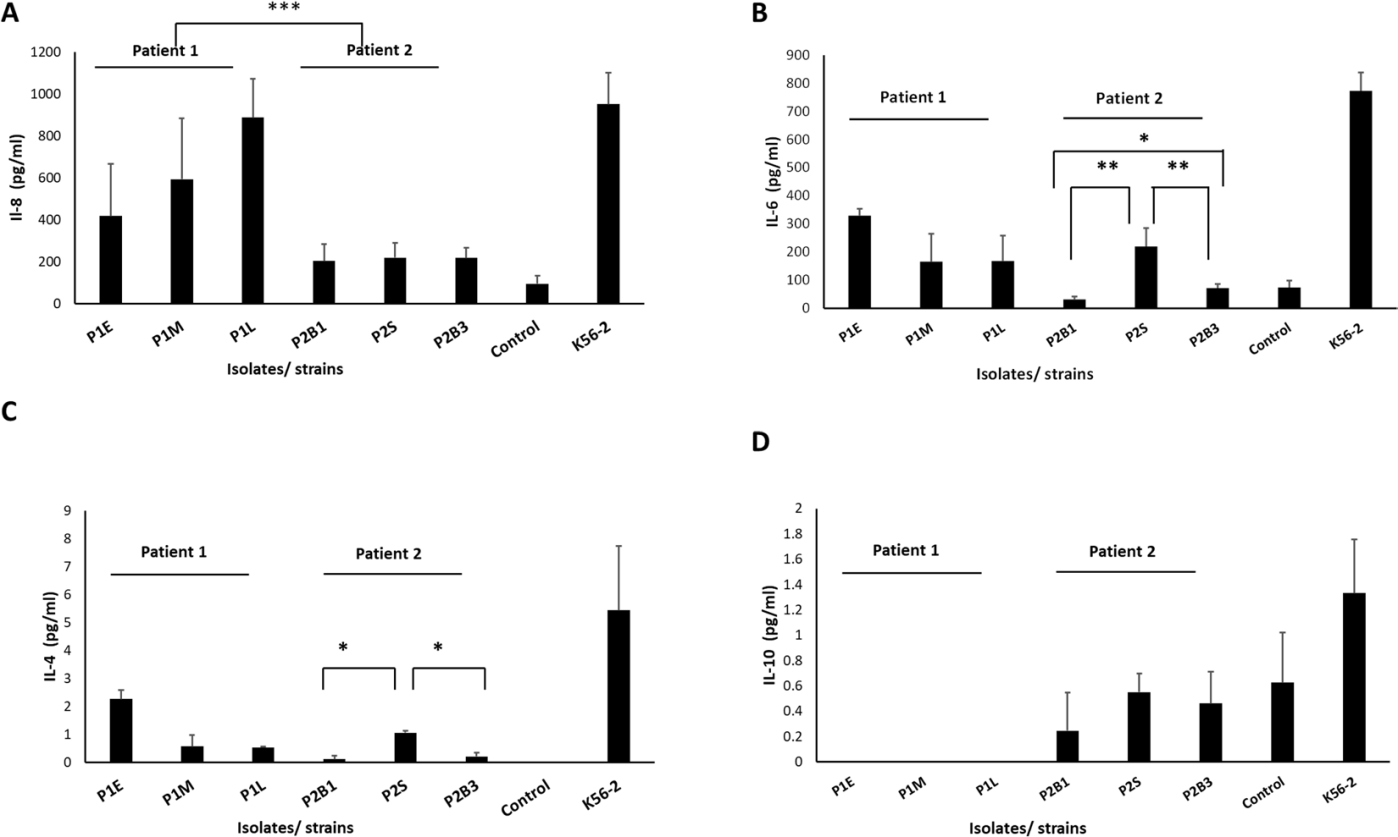
Cytokine secretion from CFBE41o- cells in response to infection with the sequential *B*. *cenocepacia* isolates (MOI 50:1). (**A**) IL-8 secretion as determined by ELISA. (**B**,**C**) Cytokine release as determined by electrochemiluminescence (**B**) IL-6; (**C**) IL-4; (**D**) IL-10. Bars represent means following three independent experiments performed in duplicate, error bars represent standard deviation. P value signifies a statistically significant difference as determined by ANOVA, *P < 0.05, **P < 0.01, *** < 0.001.

We previously demonstrated that Bcc strains promote potent IL-8 and IL-6 secretion from CF epithelial cells^34^. The alterations in proteins associated with LPS and O-antigen biosynthesis prompted us to compare proinflammatory cytokine secretions of cystic fibrosis bronchial epithelial cells (CFBE41o^−^) in response to these sequential isolates. Despite their low virulence in *G*. *mellonella*, the sequential sputum isolates induced comparable levels of IL-8 compared with *B*. *cenocepacia* K56-2 strain (Fig. 3A) (P = 0.111). A trend for increased IL-8 chemokine induction by P1 isolates relative to time of colonisation (P = 0.058) was observed, consistent with the increase abundance of proteins involved in LPS and O-antigen biosynthesis. The blood and sputum isolates from P2 induced two- to four-fold less IL-8 than those induced by P1 isolates (Fig. 3A) (P = 0.001). Overall secretion of a panel of nine cytokines was low relative to *B*. *cenocepacia* K56-2 positive control. Comparable IL-6 secretion was observed in response to the sequential P1 isolates; although two- to four-fold lower than that of K56-2 (Fig. 3B). Both P2 blood isolates induced 3- to 7-fold less IL-6 relative to P2S (P < 0.01). Despite this, P2B3 induced more IL-6 than P2B1 (P < 0.05) (Fig. 3B). IL-4 induction by all isolates in CF cells was low (≤2 pg/ml) but within the dynamic range (0.02 to 154 pg/ml) and both P2 blood isolates induced lower IL-4 than the P2S isolate (P < 0.05) (Fig. 3C), consistent with IL-6 induction. Low levels of the anti-inflammatory cytokine, IL-10 were detected in all P2 isolates (0.24 to 0.55 pg/ml; limit of detection 0.03 pg/ml); while IL-10 was undetectable in response to P1 isolates (Fig. 3D). Secretion of the remaining six cytokines (IFN-γ, TNF-α, IL-12p70, Il-13, IL-1b and Il-2) were not significantly elevated over media controls in response to any of the isolates.

#### Susceptibility of P2 isolates to the bactericidal properties of serum

**Figure 4:**
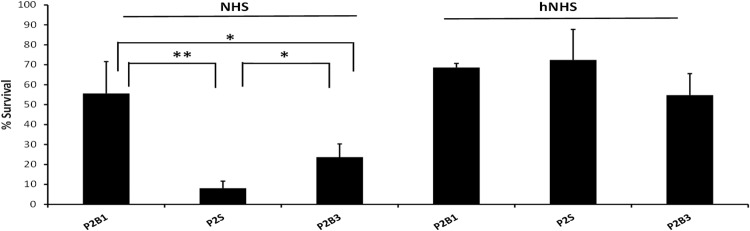
Serum resistance of the P2 blood and sputum isolates on three independent occasions. Bars represent % survival of the bacteria after treatment with 30% normal human serum (NHS) or heat inactivated normal human serum (hNHS). Data represents % bacterial survival (CFU) relative to initial bioburden. Error bars represent the standard deviation of the mean. P value signifies a statistically significant difference between the isolates as determined by ANOVA (*P < 0.05, **P < 0.01).

In the bloodstream bacteria come in contact with the bactericidal components of serum. Serum bactericidal assays showed that P2S was more susceptible to serum killing relative to P2B1 (6.9-fold, P < 0.01) or P2B2 (2.9-fold, P < 0.05), indicating a bacterial response between these environments to overcome serum killing (Fig. 4). Heat inactivated serum, allowed increased survival of P2S (P < 0.005) and P2B3 (P < 0.05), demonstrating that heat sensitive components of serum contribute to serum resistance.

#### Exopolysaccharide (EPS) production and motility in sequential isolates

Previous studies have shown differences in motility and EPS production in sequential isolates of *P*. *aeruginosa*, *B*. *cenocepacia* and *B*. *multivorans*^22,35,36^. All P1 isolates were non-mucoid compared to *P*. *aeruginosa* positive control, PA5080, indicating no EPS-associated adaptation occurred in the P1 sputum isolates over 61 months of infection. P2B1 was mucoid and both later isolates (P2S and P2B3) lost mucoidy, indicating an adaptation to a non-mucoid phenotype (Supplementary Information Fig. S2). All six ST867 isolates were non-motile for swimming, swarming and twitching compared to *P*. *aeruginosa* (data not shown).

#### Δlxa-deletion mutant shows greatly impaired host cell attachment

**Figure 5:**
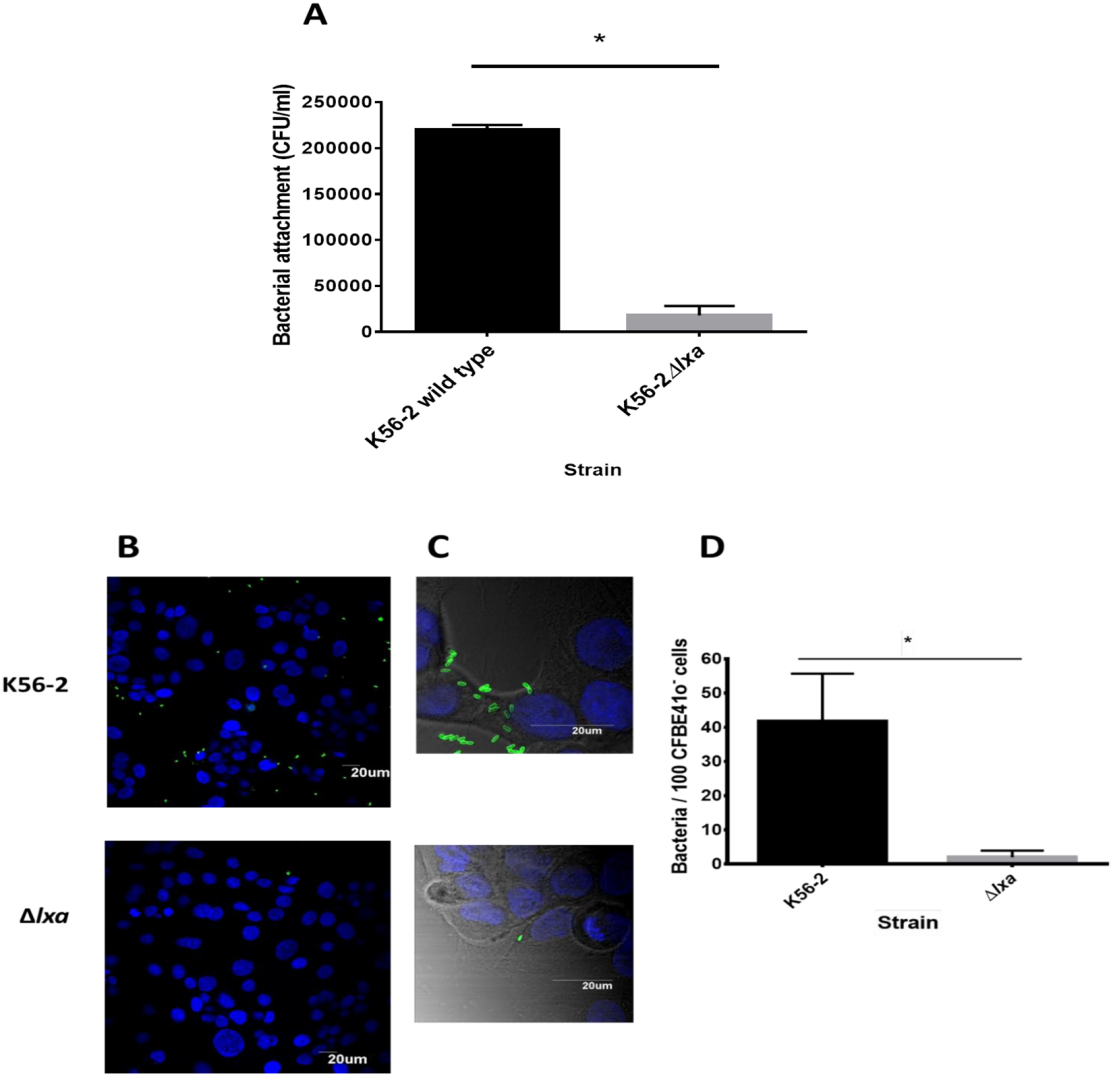
Attachment of K-562 and *Δlxa* mutant to CFBE41o^−^ cells. Adhesion of wild type *B*. *cenocepacia* strain K56-2 and *Δlxa* mutant strain to CFBE41o^−^ cells was determined by microbiological plating (**A**) and confirmed by confocal microscopy (**B**,**C**). (**A**) Mean CFU adhered per well to CFBE41o^−^ cells as determined in three independent experiments. Error bars represent standard deviation. *Statistically significant difference relative to K56-2 strain as determined by unpaired t-test, p < 0.001. (**B**) Representative confocal images of bacteria labelled with a rabbit anti-Bcc antibody and detected with secondary FITC-conjugated anti-rabbit antibody. CFBE41o^−^ cells were counterstained with DAPI. (**C**) Independent zoomed in representative images prepared as above and superimposed on differential interference contrast imaging. (**D**) Quantification of attachment following confocal microscopy. Data represents the number of bacteria/100 cells in 10 randomly selected fields for each strain. Error bars represent the standard error of the mean (SEM) from two independent experiments. *P = 0.0017.

Overall, only two consistent phenotype changes correlated with time of infection in the isolates from both patients: increased abundance of *lxa*-encoded proteins and our previous data showing increased host cell attachment. This prompted us to examine whether these two distinct phenotypes might be related and consequently, we examined the potential role of the *lxa*-locus in host cell interactions. An K56-2*Δlxa*-mutant strain^24^ showed a 12-fold reduction in attachment to CFBE41o^−^ cells relative to its parent K56-2 strain (Fig. 5A), which was confirmed by confocal microscopy (Fig. 5B,C). This suggests that at least one or more of the proteins encoded within the 50-gene cluster is either directly involved, or regulates proteins involved, in host cell attachment.

## Discussion

Genetic flexibility of Bcc isolates in CF infection has been previously reported^20,28^. Among the SNVs identified, 12 were also identified in a separate group of three sequential isolates^28^, indicating common mechanisms of adaptation during CF infection. These included genes encoding siderophore synthesis or receptors orbA, orbK, BCAL1345; transcriptional regulator genes: BCAM1722, BCAM2452, BCAS0007; antibiotic resistance genes, BCAM1362; BCAM216 and BCAM21685; gyrA and gspD. Although the WGS analysis did not show many SNVs in the LXA locus, the consistent increased abundance of LXA-encoded proteins across both sets of patient isolates may be associated with one or more of the many mutations in regulatory protein genes. Many of the proteins which were undetectable in one or more of the isolates correlated to frameshift mutations in the specific isolate affected Table S2), which probably resulted in disrupted protein expression, including BCAL2037, BCAL2444, BCAL2605, BCAM0871, BCAM0881, BCAM1395 and BCAS0081.

Despite a *mutS* mutation in the last P1 isolate and all P2 isolates and the abundance of SNVs in regulatory genes in these isolates, the phenotypes investigated appeared remarkably stable in this group of sequential isolates in contrast to other studies in Bcc^20^ and *P*. *aeruginosa*^37^. We have examined five phenotypes across these sequential isolates (virulence in *G*. *mellonella*, cytokine stimulation, motility, mucoidy and, previously, host cell attachment) and host cell attachment was the only phenotype to consistently alter with time in both sets of isolates. The increased epithelial attachment of *B*. *cenocepacia* ST867 in the isolates from both siblings suggests that this may be a general strategy facilitating survival in the host, avoiding clearance and contributing to the challenges of Bcc eradication once chronic infection is established. Increased host cell attachment has also been shown in chronic *B*. *multivorans* isolates^29^ and may also explain the previously observed increased bacterial invasion among other sequential *B*. *cenocepacia* isolates^38^. We therefore performed a proteomic analysis on six selected sequential isolates in order to identify a common mechanism which may mediate the niche adaptation associated with increased host cell attachment.

Among the altered proteins that could potentially mediate this increased host cell interaction is the BON-protein (BCAM0280), which was substantially elevated with time of colonization in both individuals’ isolates. This phospholipid-binding protein is associated with osmotic shock protection and phospholipid- or host-interactions^39^, but has not previously been linked with host cell attachment. Studies are on-going to evaluate this protein and its role in pathogenesis. The 2-fold increase in TAA BCAM0219 in the sequential P1 isolates may contribute to the increased host cell attachment of later isolates. Two TAAs in the same cluster as BCAM0219, (BCAM0223 and BCAM0224) were both previously implicated in epithelial cell adhesion^40,41^. Increased expression of this TAA (and BCAM223) was previously shown in *B*. *cenocepacia* sequential isolates^42^. Other cell-surface alterations that may contribute to the increased adherence of the sequential P1 isolates include a protein associated with outer membrane biogenesis (BCAL3149). However, neither BCAM0219 nor BCAL3149 were consistently upregulated over time of colonization in the P2 isolates, suggesting that while they may contribute to increased lung cell attachment, these are unlikely to be part of a common mechanism of enhanced host cell interaction over time of colonization. Similarly, OmpA, OmpW and putative fimbrial usher protein were increased in abundance in the later P2 isolates and could contribute to enhanced adhesion, but they are unlikely to be part of a general mechanism of increased attachment over time due to the lack of alteration in P1 isolates over time. Ideally inclusion of a sputum isolate prior to the first blood isolate in P2 would have been helpful, however none was available. However, the parallel observations in the three sequential P2 isolates support the time-related alterations observed in P1.

A consistent and unexpected finding was the time-related increase in both sets of isolates of 40% of the proteins encoded by the *lxa*-locus which is associated with *B*. *cenocepacia* survival under oxygen limitation^24^. This gene cluster encodes six USPs in addition to proteins predicted to be involved in metabolism, electron transfer and regulation and probably provides *B*. *cenocepacia* with a fitness advantage under hypoxic conditions. The blood isolates were most likely adapted to hypoxia whilst in the lungs and this adaptation was maintained in the bloodstream. The fact that significantly elevated abundance of 19 proteins encoded by this *lxa-*locus was evident in sequential *B*. *cenocepacia* isolates from both individuals over time of infection indicates a concerted response to the host. This is supported by the fact that all six USPs encoded within this locus were increased over time in both sets of isolates. Interestingly, USPs are also induced under hypoxic conditions in *P*. *aeruginosa*^43^ under the regulation of the oxygen-sensing regulatory protein, Anr. Sass *et al*.^24^ identified a potential oxygen-sensing regulator in *B*. *cenocepacia* (BCAM0049) which has 43% sequence similarity to *P*. *aeruginosa* Anr^24^. BCAM0049 is a cAMP-receptor regulatory protein (CRP) which showed time-dependent increased abundance in all comparisons from both patients (Tables 2 and 3). Recently, its homologue in *B*. *dolosa*, FixK, was shown to be under the control of an oxygen-sensing two component system, FixLJ which is under positive selective pressure during CF chronic infection^44^. Although neither mutations nor alterations in abundance of FixLJ homologues (BCAL2210/BCAL2211) have been identified in these *B*. *cenocepacia* clinical isolates, BCAM0049/fixK may play a central regulatory role to the oxygen response in both species. Furthermore, the *M*. *tuberculosis* dormancy regulon, DosR, encodes 10 USPs among its 48 genes which are also upregulated during hypoxic conditions and implicated in long-term survival in anoxia^45^. The DosR regulon comprises genes with functional or phylogenetic homology to *lxa*-locus genes associated with increased protein abundance in the later isolates, such as α-crystallin (BCAM0278) and phosphofructokinase (BCAM0311), both of which were upregulated over time in all comparisons. Whether *lxa-*locus upregulation is common across other sequential Bcc isolates remains to be explored. It is likely that the increased abundance of *lxa*-encoded proteins during chronic infection in both individuals may be a competitive strategy essential for *B*. *cenocepacia* persistence in the hostile host environment. The consistent elevated abundance of *lxa-*locus encoded proteins plus other USPs co-regulated with the *lxa-*locus^24^, BCAM1495 and BCAM1500, and the potential oxygen-sensor, BCAM0049 probably contributes to niche adaptation in the CF lung. Furthermore, the *lxa*-associated proteins are the most likely candidates contributing to the increased attachment observed in both sets of isolates over time of colonization.

The bloodstream of P2 was acutely infected with Bcc, and consequently, the alterations observed between blood and sputum isolates are likely to be acute responses to the blood environment. Several adaptations were consistent in both blood isolates relative to P2S, despite being present in the bloodstream intermittently, suggesting that these were common responses to the bloodstream. The increases in larval virulence and serum resistance of P2 blood isolates relative to the sputum isolate may relate to the enhanced abundance of TAA, BCAM0219^40,41^. Likewise, expression of a complete LPS molecule has been associated with *B*. *cenocepacia* survival in the presence of serum^46^. Reduced abundance of four proteins involved in LPS synthesis in P2 blood isolates over 18 months, may contribute to the reduced serum resistance of P2B3 relative to P2B1 isolate. In addition, the pro-inflammatory properties of lipid A would suggest that reduced abundance might benefit *B*. *cenocepacia* blood isolates *in vivo*^47,48^. Inflammation is a significant problem in CF, leading to tissue damage. The lower pro-inflammatory cytokine responses elicited by the blood isolates relative to the sputum isolate from P2, suggests adaptations to avoid host detection, facilitating bloodstream infection. The increase in Mpl abundance, in P2S relative to blood isolates, may cause substantial differences in cell surface between these isolates, which might partially contribute to the reduced IL-6 responses of blood isolates. Overall, there seems to be a concerted effort to avoid clearance from the blood, with several proteins involved.

The reduced abundance of iron acquisition proteins, including five involved in synthesis of the siderophore, ornibactin, in the blood isolates relative to P2S are likely due to the reduced availability of iron in the lung relative to blood. These findings are consistent with a recent report that OrbA, among other iron acquisition proteins, was upregulated when *B*. *cenocepacia* was cultured under iron-restricted conditions. The reduced abundance of siderophore-associated proteins in blood isolates may represent a switch towards utilisation of alternative iron sources in blood, including haemin and ferritin, which Bcc use very effectively^49^. Given that iron acquisition proteins have been identified as virulence factors in Bcc^49,50^ lower levels of these proteins by *B*. *cenocepacia* blood isolates may also benefit the organism in evading host detection in blood. Increased iron metabolism by *P*. *aeruginosa* in the lung epithelium has been implicated in increased production of free hydroxyl-radicals, contributing to pulmonary damage and host response^51^.

There was no significant change in virulence in the G. *mellonella* model observed in the isolates over time of infection, in contrast with *P*. *aeruginosa*^15,21^. Interestingly, the virulence factor, ZmpA, was not detectable in P1E or P1M but was detected in P1L, indicative of increased expression over time, while T6SS proteins which are also associated with virulence showed reduced abundance in later isolates. These and many other factors can impact on virulence, hence the lack of any observed alteration in virulence in *G*. *mellonella* may be a consequence of alterations being too subtle to be identified in this model.

It is clear that *B*. *cenocepacia* ST867 adapts over time of infection by upregulating *lxa-locus*-encoded proteins and increasing epithelial cell attachment *in vitro*. These were the only consistent time-dependent alterations across both patients’ isolates and may provide a mechanism by which *B*. *cenocepacia* chronically persists, contributing to the challenge of Bcc eradication from CF patients. Although K56-2 is a different strain (which is regularly examined as it is more amenable genetic manipulation) and consequently direct extrapolation is limited, the dramatic impairment in attachment of the Δ*lxa* mutant strain strongly suggests that this gene cluster is involved (directly or indirectly) in host cell attachment. In addition, this was an *in vitro* study and increased attachment to host epithelial cells would need to be validated *in vivo*. There is limited data on Bcc attachment *in vivo* or *ex vivo*. An in-depth study of lungs of 21 patients colonised with either *P*. *aeruginosa*, Bcc or both, clearly demonstrated that while *P*. *aeruginosa* was located in intraluminal mucus and not adherent to airway epithelial surfaces, *B*. *cenocepacia* behaved differently and appeared to grow as single cells or small clusters in macrophages, within mucus, or occasionally within epithelial cells^52^.

Sass *et al*.^24^ also predicted that the lxa locus was also involved in metabolism, transport and electron transfer. Consistent with this, over 249 individual proteins that were classified as having a metabolism function showed altered abundance in the sequential isolates, with 52% of these showing reduced abundance. Among these proteins were 72 proteins classified as being energy metabolism and/or energy conversion proteins; 51 proteins involved in amino acid metabolism; 21 proteins classified as being involved in carbohydrate metabolism; 30 proteins involved in lipid metabolism and 10 proteins involved in fatty acid metabolism (Supplementary Information Table S4) and highlight a substantial alteration in Bcc metabolism over time of chronic colonisation, some of which may be linked to the upregulation of the lxa locus.

More studies are required to determine if activation of the *lxa-locus* is common among larger cohorts of Bcc patient isolates. Furthermore the sputum from P2 showed markedly reduced virulence in *G*. *mellonella*, increased inflammatory properties, and enhanced serum susceptibilities relative to the blood isolates from the same patient, highlighting that Bcc also shows adaptive responses to the blood environment, even in the short term. The mechanisms behind the phenotypic differences in bacterial isolates at a given time point can be complex, but understanding the key adaptations that differentiate Bcc blood and sputum isolate types should contribute to the prevention of potentially life threatening bloodstream infections in these patients. Overall, these adaptations are likely to be beneficial during infection, contributing to bacterial survival during chronic infection in CF.

## Methods

### *B. cenocepacia* sequential isolates

Sequential isolates from two adult siblings with CF with the same unique sequence type (ST867)^23^ were investigated. In order to carry out an in-depth analysis, the earliest available isolate from P1 (P1E) was compared with two randomly selected sputum isolates from that patient (P1M (middle) and P1L (late)) with an overall time span of 61 months. The earliest available isolate from P2 was a blood isolate (P2B1) which was compared with the last available blood peripheral isolate (P2B3, 16 months later) together with a sputum isolate (P2S) from P2 (Supplementary Information Table S1). The blood infections were intermittent, so these isolates were considered to have adapted to the CF lung prior to colonising the peripheral blood. All isolates were routinely cultured overnight in Luria Bertani (LB) broth (Sigma-Aldrich) at 37 °C.

### Ethics statement

The isolates analysed in this study were collected during routine clinical practice and as such this study was exempt from ethical review.

### DNA isolation and sequencing

Bacterial genomic DNA was isolated using a DNeasy blood and tissue kit (Qiagen) as per manufacturer’s specifications for Gram negative bacteria. A DNA sequencing library of bacterial genomic DNA was created using an Illumina Nextera XT preparation kit adding flow cell attachment and bar code through transposon insertion. These libraries were sequenced using Illumina V2 2 × 250 bp chemistry on the Illumina MiSeq Platform.

### Genome assembly and SNV analysis

Low quality sequence reads and Illumina adaptors were removed from raw fastq files using Trimmomatic^53^. De novo genome assembly of all six isolates was undertaken using SPAdes genome assembler v.3.5.0. Reads for all six isolates were individually aligned against *B*. *cenocepacia* J2315 using BWA-MEM version 0.7.10^54^. Sorting of BAM files and duplicate removal were performed with PicardTools 2.8.2. SNVs were called independently using the GATK toolkit^55^ following the best practices workflow^56^ and also SAMtools mpileup^57^. SNPs that were called by both SNP callers were considered *bona fide*. Blast ring image generator (BRIG) was used to generate visual genome comparisons in terms of similarity and coverage to the *B*. *cenocepacia* J2315 reference genome^58^.

### Phylogenetic analyses

Phylogenetic analyses were conducted in MEGA7^59^. The phylogenetic relationships of fourteen *B*. *cenocepacia* strains/isolates, including our six isolates of interest, were generated using the Neighbor-Joining method^60^ with 100 bootstrap replicates undertaken to display branch supports^61^. The input alignment was a concatenated alignment of seven housekeeping genes (BCAL0036, BCAL0289, BCAL0421, BCAL0953, BCAL1003, BCAL1861 & BCAM0991). Each of these genes were individually aligned and subsequently concatenated to give a final superalignment of 13,827 nucleotides. We removed phylogenetically uninformative sites and all positions containing gaps resulting in a final input alignment of 1508 nucleotides. All positions containing gaps and missing data were eliminated. There were a total of 1508 positions in the final dataset. Branches corresponding to partitions reproduced in less than 50% bootstrap replicates were collapsed. The evolutionary distances were computed using the Maximum Composite Likelihood method and are in the units of the number of base substitutions per site. Additional evolutionary analyses of the isolates of interest were inferred using the Maximum Parsimony (MP) method. The input character data corresponded to SNP data located in this analysis. The MP tree was obtained using the Subtree-Pruning-Regrafting (SPR) algorithm with search level 1 in which the initial trees were obtained by the random addition of sequences (10 replicates). Branch supports were inferred using 100 bootstrap replicates.

### Proteomic analysis of sequential *B. cenocepacia* isolates

Bacterial proteins were isolated as previously described^62^ from isolates which had been cultured overnight in LB broth at 37 °C, pelleted at 2778 × g for 15 min and pellets resuspended in 0.25% (v/v) Triton X-100, 40 mM Tris pH 7.8, containing protease inhibitor cocktail (Roche) and sonicated on ice. Lysates were centrifuged 10,000 x g for 15 min, methanol precipitated at −80 °C overnight, centrifuged at 10,000 x g at 4°C for 30 min and proteins resuspended in 2 ml 8 M urea, 50 mM Tris-HCl pH8. Reduction, alkylation, dialysis and tryptic digestion were performed according to published methods^63^. Peptides (20 µl) were dried and resuspended in 0.5% trifluoroacetic acid (20 µl), sonicated and purified on C18 Zip-tips (Millipore) prior to mass spectrometry on a Q-Exactive hybrid quadrupole orbitrap LC-MS/MS (Thermo Scientific)^64^. Three biological replicates and one technical replicate of each digested sample were analysed.

MS files were analysed against the *B*. *cenocepacia* J2315 protein database (Uniprot). Comparative proteome abundance and data analysis were performed using MaxQuant software (Version 1.3.0.5)^65^, with Andromeda for database searching and Perseus (Version 1.4.1.3) for organisation and statistical analysis^66^ with the following settings: fixed modification- carbamidomethylation of cysteines; variable modifications- oxidation of methionines and acetylation of N-termini; peptide/protein false discovery rates (FDR) set at 1% based on comparison to a reverse database. The Label-Free Quantification (LFQ) algorithm was used to generate normalised spectral intensities and infer relative protein abundance. Proteins were only retained in the final analysis if detected in at least three replicates from at least one sample. Proteins with significant abundance changes (t-test, p < 0.05; fold change ≥1.5) were included in the quantitative results. Qualitative analyses were also performed to detect proteins found in at least 3 replicates of a sample, but undetectable in the comparator sample. Differentially expressed proteins were searched in the *Burkholderia* database (http://burkholderia.com/) to identify potential functions^27^. Expasy software was used to compute pIs (http://web.expasy.org/compute_pi/) and subcellular locations were determined with Psort v3.0.2 (http://www.psort.org/psortb/index.html).

### Virulence in *Galleria mellonella*

The virulence of the sequential isolates in *Galleria mellonella* larvae were determined as described previously^67^. Bacterial cultures (OD_600nm_0.6) were serially diluted in phosphate buffered saline (PBS) from 10°–10^−7^ and 20 µl aliquots injected into the haemocoel of 10 healthy larvae per group. Negative control larvae were injected with PBS (20 µl). The percentage survival was determined up to 72 h. LD_30_ (CFU required to kill 30% of larvae at 48 h) was calculated from three independent experiments.

### Cytokine secretion by CFBE41o- cells

Cytokine secretion by CFBE41o^−^ cells was determined as previously described^68^, with minor modifications. IL-8 levels were determined using an OptEIA^™^ human IL-8 ELISA kit (Becton-Dickenson). A wider range of cytokines were analysed using a chemiluminescent ELISA system, (V-PLEX^TM,^MesoScale Discovery) to detect IFN-γ, TNF-α, IL-10, IL-12p70, IL-13, IL-1β, IL-2, IL-4 and IL-6 according to the manufacturer’s instructions. Cytokines were determined in duplicate on three independent occasions.

### Assessment of sequential isolates susceptibility to the bactericidal potential of serum

Serum bactericidal assays were performed as previously outlined^41^. Blood isolates (100 µl) were added to 30% (v/v) normal human serum (NHS) (Sigma-Aldrich) or to heat inactivated NHS (hNHS). Initial bioburden was determined with 1:10 dilutions of cultures in PBS. All samples were incubated at 37 °C for 1 h (30 rpm), chilled and plated onto LB agar in duplicate. The CFU at 48 h were expressed as % survival relative to initial bioburden. Each experiment was performed three independent times.

### Real time PCR

*B*. *cenocepacia* cultures (OD_600_ 0.4–0.8) were incubated with RNAprotect^®^ (Qiagen), centrifuged and lysed with lysozyme (1 mg/ml) in 10 mM Tris-HCl, 1 mM EDTA (pH7.5) for 10 min. RNA was isolated by RNeasy procedure (Qiagen) and DNase treated with turbo DNA-free kit^™^ (Ambion) and converted to cDNA using Superscript^®^ VILO™ cDNA synthesis kit (Invitrogen). Primers (Bio-sciences) were designed using NCBI primer design tool (Supplementary Information Table S7). QPCR was performed using Power SYBR^®^ green master mix in 25 µl containing 250 ng cDNA, 300 nM forward and reverse primers. No-template controls were included on each plate and amplification performed on Applied Biosystems 7300 real-time PCR system: initial step 95 °C, 10 min; 40 cycles of 95 °C, 5 sec and 60 °C for 1 min^69^. Relative quantification (RQ) expression differences were determined using the 2^−ΔΔCt^ method.

### Bacterial attachment to human CF epithelial cells

Bacterial attachment of wild type *B*. *cenocepacia* strain, K56-2 or *Δlxa* mutant to CFBE41o^−^ cells (5 × 10^4^ cells) on coated chamber slides (multiplicity of infection (MOI) 50:1) was determined after 30 min incubation by lysing the cells with 0.5%(v/v) Triton X-100 for 20 minutes, serial dilution and plating on LB agar as outlined^70^ on three independent occasions. Confirmation of attachment was performed by confocal microscopy by fixing the CFBE41o^−^ cells in 3% paraformaldehyde and detection with rabbit anti-Bcc antibody in 1% BSA overnight at 4 °C.

### Statistical analysis

All statistical analyses were performed using Minitab Statistical software package (v15) unless otherwise stated. Analysis of host cell attachment, cytokine secretion and serum resistance data were determined by one-way ANOVA. Virulence data were analysed by comparing LD_30_ values between the isolates at 48 h by one-way ANOVA. Proteomic data were analysed by Perseus (Version 1.4.1.3)^66^ with t-tests performed for pairwise comparisons of samples (p < 0.05; fold change >1.5).

## Electronic supplementary material

Supplementary Information

Supplementary Tables
