# Supplementary material for: The involvement of the low-oxygen-activated locus of Burkholderia cenocepacia in adaptation during cystic fibrosis infection
Source: Sci Rep. 2018 Sep 6;8:13386. doi: 10.1038/s41598-018-31556-6 (PMC6127331; doi:10.1038/s41598-018-31556-6)

**The involvement of the low-oxygen-activated locus of *Burkholderia cenocepacia* in adaptation during cystic fibrosis infection.**

Louise Cullen^1^, Andrew O’Connor^1,2^, Sarah McCormack, Rebecca A. Owens^3^, Giles S. Holt^4^, Cassandra Collins^1^, Máire Callaghan^1^, Sean Doyle^3^, Darren Smith^4^, Kirsten Schaffer^5^ , David Fitzpatrick^3^ and Siobhán McClean^1,2*^

**Supplementary information**

**Supplementary Methods**

*Confirmation of WGS SNPs by Sanger Sequencing*

Genes of interest were amplified by PCR using the following primers: BCAM0292 forward primer = 5’-TTCATCCACATCCGTTCAAGG-3’; reverse primer = 5’-GTAGCTCATGACGGTTCCTTTTCG-3’. BCAL1700 forward primer = 5’-CGGATGGCTTTCGCTCTTTG-3’; reverse primer = 5’-CGACGGGATCGAACAGAACA-3’.

PCR products were confirmed as being the expected size by gel electrophoresis on a 1% agarose gel. Amplicons were purified using the QIAquick PCR purification kit from Qiagen. Purified amplicons were sequenced by Eurofins Genomics (Dublin, Ireland). Sequences from sequential isolates were aligned with each other, and to the gene sequence from Burkholderia cenocepacia J2315 (Burkholderia.com)[1], using SeaView4 software.

*EPS production.*

EPS production was determined on yeast extract mannitol (YEM) agar (0.2% yeast extract, 2% mannitol in Agar no 2) (6). After inoculation, YEM plates were incubated at 37^o^C for five days and EPS production was scored according to a method by Zlosnik *et al.* (2008) (7)*.* The scoring of non-mucoid through to frankly mucoid phenotypes was as follows: -, +, ++ and +++. Each analysis was performed on three independent occasions.

## *Motility assays*

Assays for swimming, swarming and twitching motilities were performed according to previously published methods with minor modifications (8). Swimming and swarming motilities were assessed by inoculating the surface of 0.3% (w/v) and 0.5% (w/v) LB agar plates respectively with overnight cultures using sterile toothpicks. Swimming and swarming plates were incubated overnight at 37 °C and 30 ^o^C respectively for 16-18 hours. Twitching motility was assessed using 1% (w/v) LB agar inoculated to the base of the petri dish with overnight bacterial cultures using a sterile toothpick and incubated for 16-18 hours at 37 °C. Zones of motility at the agar/petri dish interface were subsequently stained using 0.5% (w/v) Coomassie brilliant blue R250 to improve visualisation (9). The diameter of the swimming, swarming zones and twitching zones were calculated by taking an average of two perpendicular measurements. For swimming and swarming motilities strains were scored as non-motile (diameter ≤ 5 mm), motile (diameter > 5 mm and ≤ 60 mm), or highly motile (diameter > 60 mm). For twitching motility bacterial isolates were scored as non-motile (diameter ≤ 5 mm), motile (diameter > 5 mm and ≤ 30 mm), or highly motile (diameter > 30 mm). Three biological replicates were performed for each assay.

**Supplementary Figure S1**

1. BCAM0292 alignment, confirming the missense variant at position 205 (G>A) in P2B
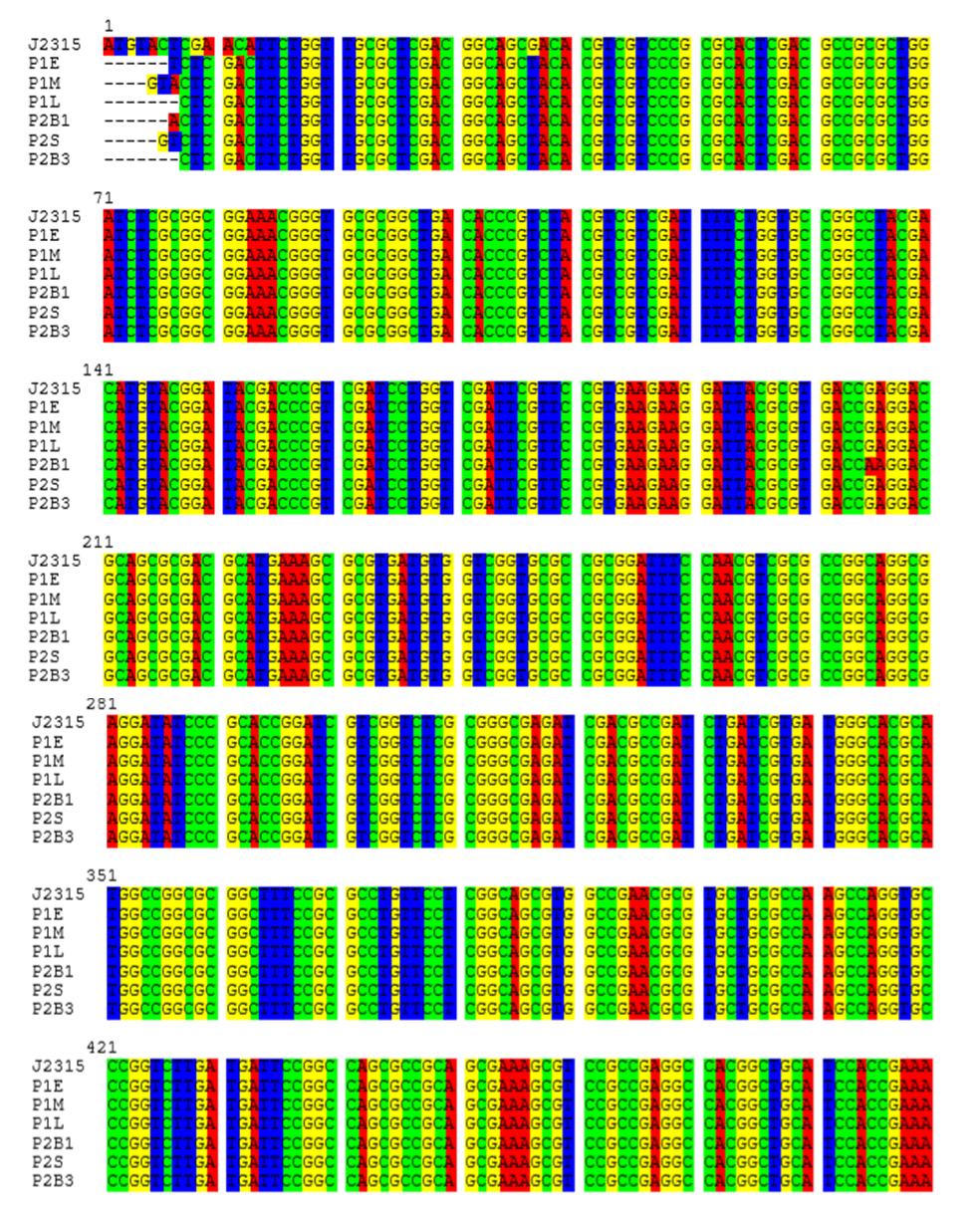


**B)** BCAL1700 alignment (first 840b), confirming a synonymous variant at position 222 (G>A) in P2B1 and P2S and a missense variant at position 789 (C>T) in P2S (Supplementary Figure 2B)


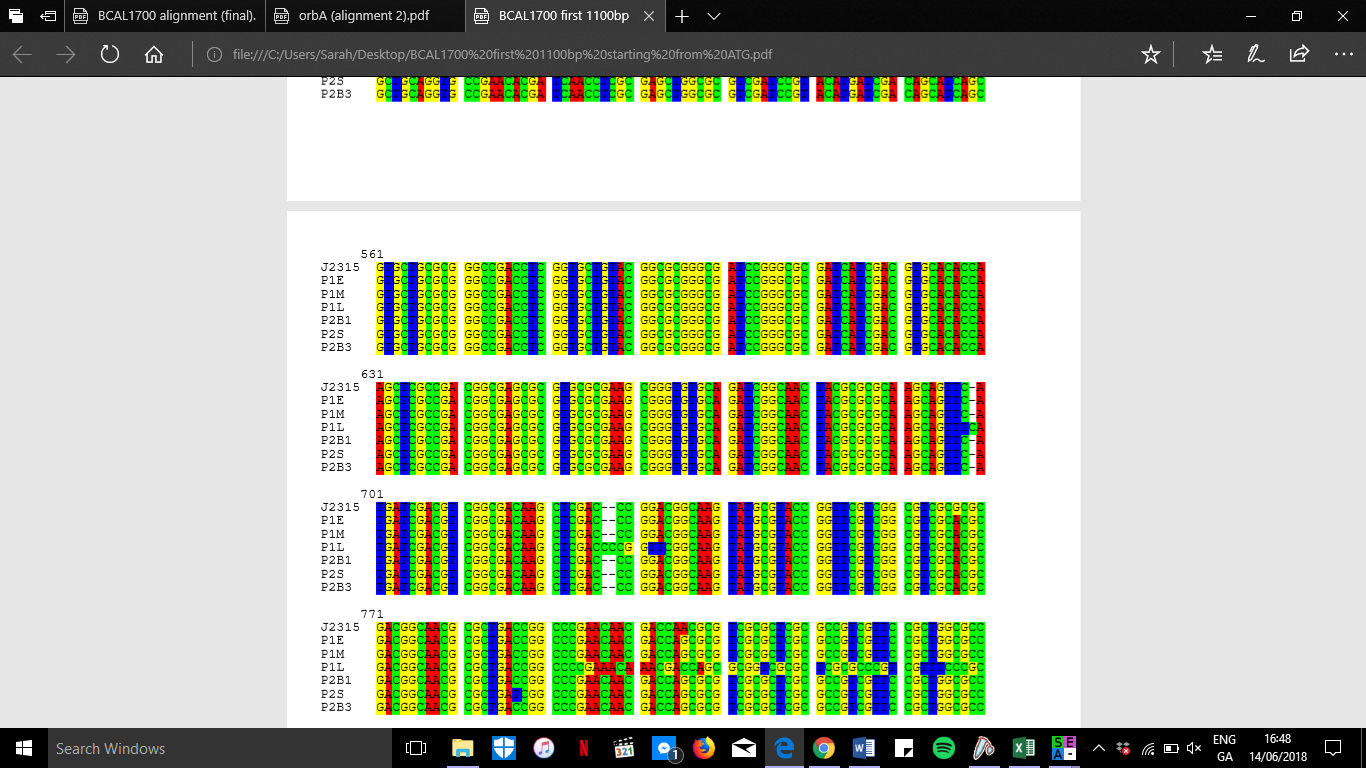

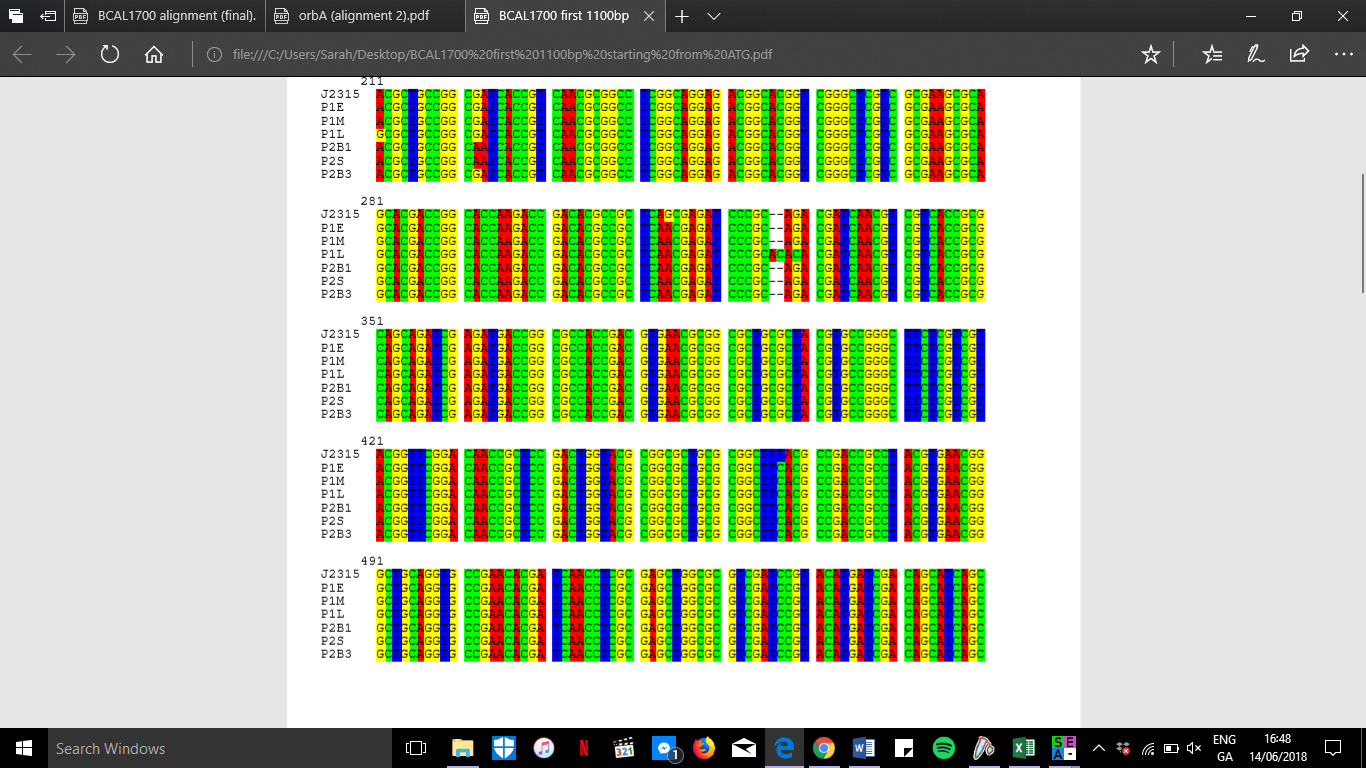

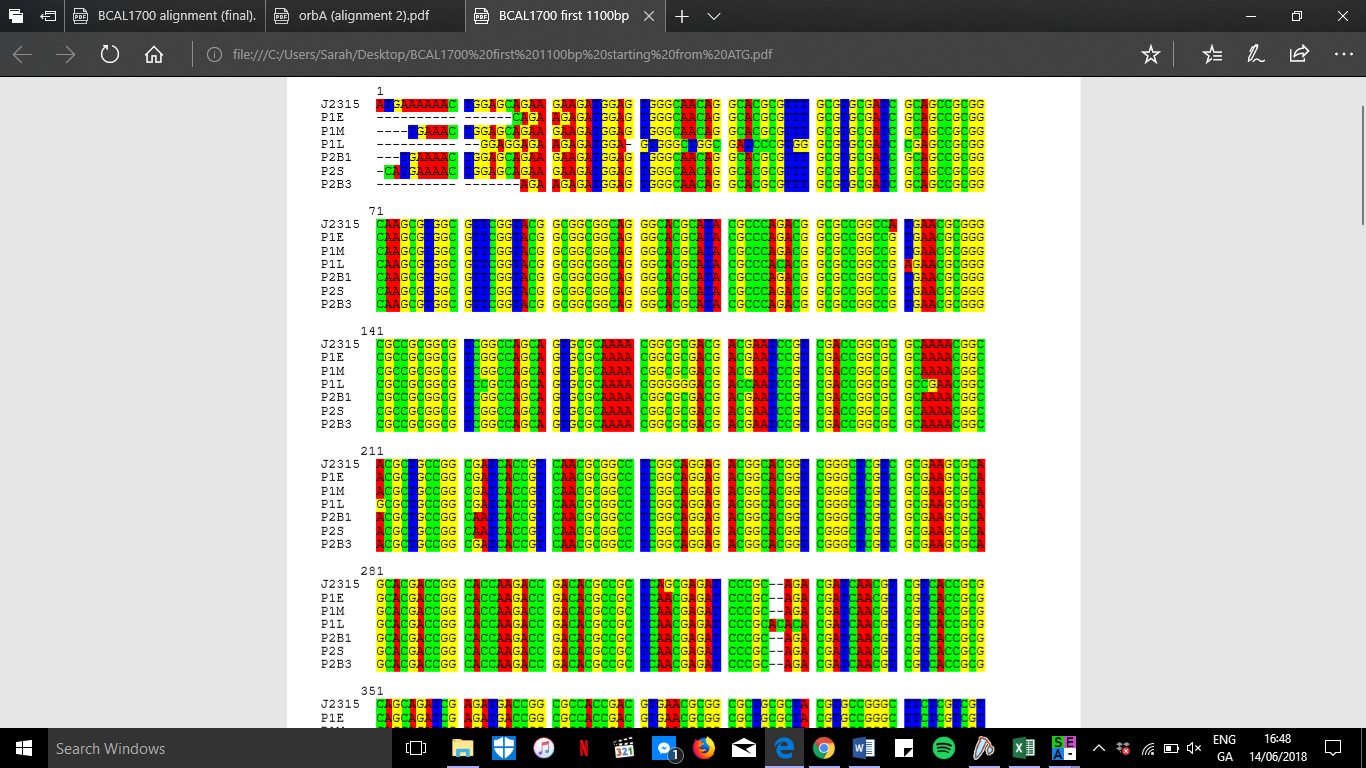


**Supplementary Figure S2**: Representative images showing the EPS production by the isolates from P2 on YEM agar after 5 days incubation at 37 ^o^C, highlighting the loss in mucoidy over the 18 month period. A) early blood, B) sputum isolate and C) late blood isolates.


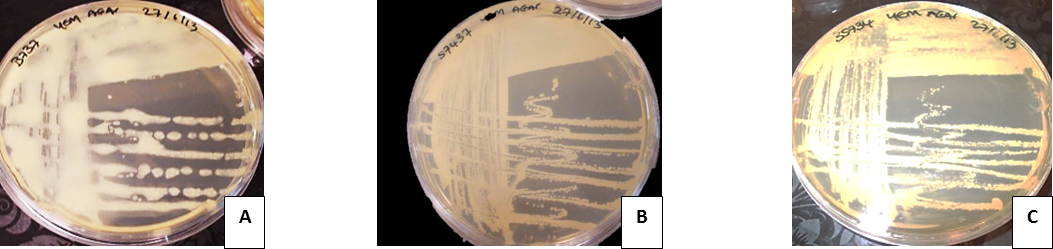

Supplement: Supplementary file 1 — Supplementary Information [file 41598_2018_31556_MOESM1_ESM.docx]
